# DEVELOPMENT AND APPLICATION OF BRAIN TISSUE BASED MULTI-OMICS PROFILE SCORES FOR ALZHEIMER’S DISEASE

**DOI:** 10.21203/rs.3.rs-9438710/v1

**Published:** 2026-04-27

**Authors:** Timur Tug, Donghai Liang, Sven Teschke, Youran Tan, Marla Gearing, Allan I. Levey, James J. Lah, Aliza P. Wingo, Thomas S. Wingo, Michael Lau, Katja Ickstadt, Anke Hüls

**Affiliations:** 1 Department of Statistics, TU Dortmund University, Dortmund, Germany,; 2 Department of Epidemiology, Rollins School of Public Health, Emory University, Atlanta, GA, USA,; 3 Gangarosa Department of Environmental Health, Rollins School of Public Health, Emory University, Atlanta, GA, USA,; 4 Department of Pathology and Laboratory Medicine, Emory University, Atlanta, Georgia, USA,; 5 Department of Neurology, Emory University School of Medicine, Atlanta, GA, USA,; 6 Department of Psychiatry, University of California, Davis, Sacramento, CA USA,; 7 Department of Neurology, University of California, Davis, Sacramento, CA USA,; 8 Alzheimer’s Disease Research Center, University of California, Davis, Sacramento, USA,; 9 Mathematical Institute, Heinrich Heine University, Düsseldorf, Germany; 10 eBay Inc., San José, CA, USA

**Keywords:** Alzheimer’s disease, multi-omics, profile scores, DNA methylation, metabolomics, neuropathology, machine learning

## Abstract

**BACKGROUND:**

Advances in omics technologies, such as epigenomics and metabolomics, provide novel insights into the biological mechanisms underlying Alzheimer’s disease (AD). However, little is known how different omics layers interact and jointly relate to AD neuropathology.

**METHODS:**

We performed a comprehensive single- and multi-omics analysis integrating genome-wide DNA methylation and high-resolution metabolomics data from 157 frontal cortex samples. We developed novel single and multi-omics profile scores (PS) for AD pathology, using a combination of machine learning, regression, and pathway analysis.

**RESULTS:**

For the ABC score (Amyloid, Braak, CERAD) the PS of DNAm outperformed metabolomics-based PS (median R†: 0.11 vs. 0.04). Combining both omics layers with the best-performing multi-omics PS yielded a partial R† of 0.15 for the ABC score independent of age, sex, race and socioeconomic factors. DNAm-specific pathways highlighted redox balance, immune activation, synaptic signaling, and lipid biosynthesis, whereas metabolomics-specific pathways emphasized inflammatory, hormonal, lipid, and energy metabolism. Notably, both omics layers converged on lipid metabolism and signal transduction as shared biological systems implicated in AD neuropathology.

**CONCLUSIONS:**

Despite limited gains in predictive accuracy, integrative pathway and network analyses of DNAm and metabolomics PS converged on lipid metabolism and signal transduction, underscoring shared biological mechanisms and the value of multi-omics approaches for biological insight rather than prediction alone.

## INTRODUCTION

Alzheimer’s disease (AD) is a progressive neurological disorder that affects millions of Americans, with approximately 6.7 million people aged 65 and older currently living with the condition [1]. AD is the fifth-leading cause of death among older adults in the United States and poses a major public health challenge [2]. The financial burden is equally staggering, with the annual cost of care for AD patients projected to reach $580 billion in 2025, a figure expected to rise substantially in the coming decades [1, 3]. As the prevalence of AD continues to rise globally due to aging populations, developing effective strategies to reduce its burden has become a critical priority. Substantial efforts are underway to create disease-modifying therapies targeting the molecular mechanisms of AD [4, 5], but the complex and multifactorial nature of the disease has posed significant challenges.

Advances in high-throughput omics technologies have provided new insights into the biological pathways and signatures underlying AD, offering promising avenues for innovative therapeutic strategies [6, 7]. DNA methylation (DNAm) and metabolomics analyses have emerged as powerful tools for exploring AD etiology. These approaches offer complementary insights into AD’s complex pathophysiology by capturing information on epigenetic changes and metabolic dysfunction—both recognized as core features of the disease [8]. DNAm, a key epigenetic mechanism, is closely linked to AD, with global hypomethylation, gene-specific methylation changes, and interactions with neuroinflammation, aging, oxidative stress, and environmental factors contributing to disease risk and progression. As the downstream product of the gene transcription and gene-environment interaction, metabolomics involves studying small molecules (metabolites) in biological systems, which can reveal changes in metabolic pathways associated with AD. Perturbations in metabolomics pathways that have been reported in association with AD include dysregulation in energy metabolism, lipid profiles, amino acid pathways, oxidative stress, and gut-brain axis interactions [9–13]. However, little is known how differences in DNAm and metabolomics interact and jointly influence the development of AD. Integrating DNAm and metabolomics through a multi-omics approach could illuminate shared biological pathways that may be particularly important for understanding AD etiology.

To address this knowledge gap, we conducted a comprehensive multi-omics analysis integrating genome-wide DNAm and high-resolution metabolomics data from 157 prefrontal cortex tissue samples of brain donors with varying stages of AD pathology, assessed with Braak staging, CERAD (**C**onsortium to **E**stablish a **R**egistry for **A**lzheimer’s **D**isease) scoring, and the comprehensive ABC score (**A**myloid, Braak, **C**ERAD) [14, 15]. Specifically, we calculated multi-omics profile scores (PS) integrating DNAm and metabolomic data, providing a holistic understanding of AD neuropathology by capturing both epigenetic and metabolic contributions. Our analysis framework not only identifies CpG sites and/or metabolomic features predictive of AD neuropathology levels but also elucidates mechanistic underpinnings by evaluating and comparing biological pathways enriched among the selected CpG sites and/or metabolomic features. The multi-omics framework developed in this study highlights the potential of combining epigenetic and metabolomic data to deepen our understanding of AD pathophysiology. This study extends the current state of research through several innovative approaches. We proposed novel brain tissue-based multi-omics profile scores for AD, which integrate DNA methylation and metabolomics to better predict the neuropathological changes of the disease. Methodologically, advanced machine learning techniques such as Random Forest, Elastic Net and Boosting are used to efficiently analyze high-dimensional data and generate robust profile scores. These novel analysis strategies and methods and a comprehensive pathway analysis allow a more precise identification of relevant biological mechanisms.

## METHODS

### Study design

The Emory Goizueta Alzheimer’s Disease Research Center (ADRC) established a brain bank to support Alzheimer’s research, primarily enrolling research participants and patients clinically diagnosed with AD by Emory physicians. By the third quarter of 2020, the brain bank included 1,011 donors. Genome-wide DNAm and metabolomics profiling were conducted on 161 samples from donors deceased after 2007, with 159 samples passing quality control. All 161 donors had complete data for key covariates (e.g., age of death, race, sex and educational attainment) and outcome variables (e.g., ABC score, Braak Stage, CERAD). Informed consent and Institutional Review Board-approved protocols governed the research.

### Assessment of AD neuropathology

The ADRC conducted comprehensive neuropathologic evaluations on all donor brains using established research protocols and diagnostic criteria [14]. These assessments, performed by experienced neuropathologists, involved a variety of stains and immunohistochemical techniques, along with semi-quantitative scoring to evaluate AD and related neuropathologies in various brain regions [16]. AD neuropathology in this project was measured using the Braak staging, CERAD score, and ABC score - each assessing different aspects of disease progression. Braak Stage classifies the spread of neurofibrillary tangles (NFTs) tau-containing protein deposits, a hallmark of AD - in the brain across six stages [17]. Early stages (I and II) indicate NFTs in trans entorhinal regions, while intermediate stages (III and IV) involve limbic regions, and later stages (V and VI) show NFTs spread throughout cortical areas. Higher stages reflect more extensive disease progression and broader NFT distribution in the brain [17]. The CERAD score evaluates the density of neuritic plaques, mainly composed of beta-amyloid, and categorizes them into four levels: none, sparse, moderate, and frequent [18]. These plaques are a primary indicator of AD, with higher CERAD scores signifying a greater accumulation of plaques, reflecting more advanced amyloid pathology [18]. The ABC score combines data from the Braak and CERAD scores with the Thal phase, which describes the spread of amyloid plaques in the brain. Thal staging ranges from phase 1 (amyloid in subcortical areas) to phase 5 (widespread distribution across the brain) [19]. The ABC score synthesizes information on NFT spread and amyloid plaque density into a single assessment of AD pathology, categorizing it into four levels: none, low, intermediate, or high [15]. This score provides a more comprehensive evaluation of AD severity and helps to indicate the overall stage of the disease.

### Genome-wide DNA methylation

DNA was extracted from fresh-frozen prefrontal cortex tissues in 161 samples using the QIAGEN GenePure kit. DNAm was assessed using the Illumina Infinium MethylationEPIC BeadChips, processed in batches of 167 prefrontal cortex samples, which included six replicates. The raw intensity files were converted into a dataset containing beta values for each CpG site. These beta values were calculated as the ratio of the methylated signal to the total signal (methylated plus unmethylated), ranging from 0 to 1 on a continuous scale.

Preprocessing and quality control were conducted in R (v4.2.0) [20] using a validated quality control and normalization pipeline as previously described [21]. Out of the initial samples, 159 passed the quality control checks. After excluding single nucleotide polymorphism (SNP) probes, XY chromosome probes, and other low-quality probes, 789,286 CpG sites were retained for further analysis.

The final DNAm beta values were normalized to minimize probe-type differences and adjusted using ComBat to account for batch effects prior to downstream analyses [22]. Cell-type proportions (neuronal vs. non-neuronal cells) for each sample were estimated using the latest prefrontal cortex reference database and the R package *minfi* [23–30].

### High-resolution metabolomics

High-resolution metabolomic profiling of prefrontal cortex tissue was conducted using liquid chromatography coupled with high-resolution mass spectrometry (LC-HRMS) following established protocols [31–34]. Each sample was analyzed in triplicate with two complimentary chromatographic methods: hydrophilic interaction liquid chromatography (HILIC) for polar metabolites and reverse-phase chromatography (C18) for less polar compounds to enhance the coverage of feature extraction. Detected signals were characterized by accurate mass-to-charge ratio (m/z), retention time (RT), and ion intensity [35].

Raw data were converted to .mzML format and processed with apLCMS and xMSanalyzer for peak detection, alignment, feature quantification, batch correction, and quality filtering [36, 37]. Metabolomic features were further screened before being included in the final analysis based on strict criteria: detected in >15% of samples, median CV <30%, and Pearson correlation ρ > 0.7 among technical replicates. Data were averaged across replicates with non-zero intensities and log2-transformed for statistical analyses. Finally, we included 20,051 features at the HILIC and 15,297 features at the C18, a total of 35,348 features.

### Covariates assessment

All models were adjusted for *a priori* selected covariates based on the literature, which include demographic and socioeconomic factors. Individual-level characteristics included sex, race (Black vs. White), educational attainment (high school degree or less, college degree, graduate degree) and age at death. The Area Deprivation Index (ADI) served as a proxy for neighborhood socioeconomic status, based on the 2015 Census Block Group data [38], indicating socioeconomic disadvantages in income, education, employment, and housing. The postmortem interval (PMI) refers to the time elapsed between a person’s death and the collection of biological samples, such as tissue or blood. PMI is crucial in research involving post-mortem samples because it can impact on the quality and stability of molecular markers, including DNAm and metabolomics.

### Statistical analysis

Following a similar analysis pipeline as established for polygenic risk scores [39], we developed single- and multi-omics PS based on metabolomics (PS_Metabolome_) and DNAm (PS_DNAm_) data, to predict AD neuropathology. A PS represents a weighted sum of selected variables:

(1) $$PS=\sum_{k=1}^{K}\beta_{k}m_{k},$$

where *K* is the number of selected variables (e.g., metabolites and/or CpG sites), *β*_*k*_ the weight assigned to the *k*-th feature and *m*_*k*_ is the actual value of the *k*-th feature.

**Figure 1.**
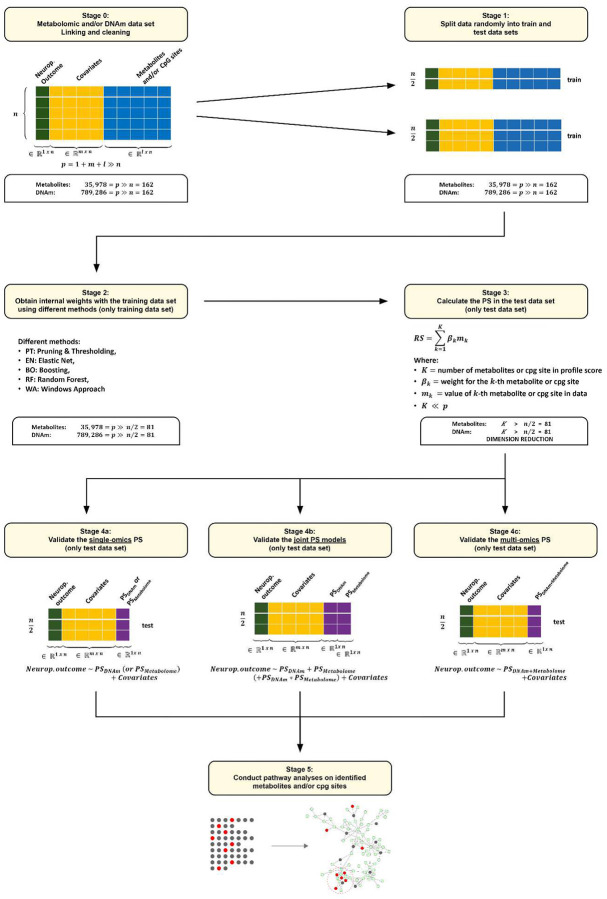
Overview of the statistical procedure for determining the weights and for calculating and validating the individual PS. First, the respective data are prepared and linked to relevant demographic covariates (age at death, race, gender, postmortem interval, Education and Area Deprivation Index) and outcomes (neuropathologic scores such as ABC score, Braak Stage and CERAD score). Then the data is split into training and test data. Various methods are used to determine the required internal weights based on the training data set. The following methods are used here: PT: Pruning & Thresholding, EN: Elastic Net, BO: Boosting, RF: Random Forest, WA: Windows Approach and combinations of these methods. The PS is now determined on the test data by means of the specific weighting of the respective PS. We validated the PS by regression against the outcomes for the single-omics PS (for each data set) and the multi-omics PS (for both data sets in one model optional with an interaction term). Finally, an optional metabolic pathway analysis using the CpG-sites (DNAm) or features (metabolomics) identified by the respective PS can be performed to evaluate relevant biological pathways.

The analytic pipeline is outlined in Figure 1 and includes the following steps that are described in the following section: Stage 0 – DNAm and/or Metabolomic dataset linking and cleaning; Stage 1 – Split data randomly into training and test datasets; Stage 2 – Estimate the weights in the training dataset using different regression and machine learning methods (PT: Pruning & Thresholding, EN: Elastic Net, BO: Boosting, RF: Random Forest, WA: Windows Approach); Stage 3 - Calculate the PS in the data dataset; Stage 4 - Validate the single- and multi-omics PS in the test data; Stage 5 - Conduct pathway analyses on identified CpG sites and/or metabolites.

#### Stages 0 and 1 – Data preparation and splitting into training and test data

Once the data sets are cleaned and merged (Stage 0), they are randomly split into training and test sets to prevent overfitting by ensuring that the models are tested on independent data. The training set is used for model building and feature selection, while the test set is reserved for evaluating model performance. In our analysis we split the data evenly into training and test data. However, with larger sample sizes, allocating a greater proportion to the training set can enhance the model’s predictive accuracy (see [39] for related recommendations for polygenic scores). We repeated this process 10 times with 10 different seeds (10 iterations) to provide a robust evaluation of our model performance. Since some of the methods described in Step 2 do not allow for missing values, we used Random Forest as implemented in the R package missRanger to impute missing values in the metabolome data set [40]. For the multi-omics PS, the DNAm and metabolites beta values were transformed into z-scores to ensure that both omics datasets are on the same scale before calculating one PS for the combined dataset.

#### Stage 2 - Feature selection and weight calculation in the training data

In the training data, variable selection and weight calculation are performed using five different regression and machine learning approaches: Pruning & Thresholding (PT), Elastic Net (EN), Boosting (BO), Random Forest (RF) and Windows Approach (WA) using cross leverage scores. This step identifies the most informative CpG sites and/or metabolites related to neuropathological outcomes. A detailed description of the methods can be found in the Supplementary Materials.

Pruning and thresholding (PT) are complementary techniques to simplify high-dimensional datasets by reducing redundancy and focusing on the most relevant variables. Pruning removes highly correlated or redundant variables, decreasing multicollinearity and computational demands while retaining predictive accuracy. For the pruning step, we applied the agglomerative (bottom-up) clustering approach [41] using the R packages ClassDiscovery [42], flashClust [43] and cluster [44]. Complete-linkage clustering was used to define the distance between two clusters as the maximum distance between any pair of points, and the Pearson correlation was used as the distance metric. The analyses generate different numbers of clusters with different numbers of CpG sites/metabolomic variables. A single representative is then drawn from each cluster and used for pruning. Thresholding applies to a predefined cutoff (e.g., p-value threshold) to filter out weak associations and retain only significant variables. We used ordinal logistics regression models (adjusted for covariates) to estimate associations between each and the neuropathology markers. Only CpG site and/or variables with a p-value < 0.05 were included in the PS.

Elastic Net (EN) is a regularized regression technique that combines the penalties of Lasso (L1) and Ridge (L2) to select variables and assign weights. To perform the analysis, we used the R package ordinalNet [45].

Boosting (BO) combines several weakly predictive models to improve the prediction accuracy [46]. We used an extended version of the Boosting method proposed by [47] to allow for ordinal outcomes. The approach considers the response variable as an ordered factor and computes thresholds to distinguish between categories. The boosting process iteratively updates coefficients, computes gradients, and adjusts predictions, resulting in a final model with optimized parameters and scaling corrections.

The Random Forest (RF) algorithm operates by creating multiple decision trees using bootstrap sampling and feature subset selection. The final prediction is then aggregated across all trees, using majority voting for classification or averaging for regression, ensuring robust and accurate predictions. We obtained variable importance measures (VIMs) within the RF fitting process using the R package ranger [48, 49]. This method performs an automatic selection of variables, and the VIMs were used as weights to calculate the PS.

The sliding windows approach (WA) involves analyzing variables within specific “windows” or genomic regions. For each window, cross leverage scores are calculated for each variable based on matrix decomposition techniques. These scores quantify the association of the variables, their interactions and the outcome. We select those *q* variables with the absolute highest cross leverage score. This method is described in more detail in [50] with an R code in the corresponding Supplementary Materials.

#### Stage 3 - Profile score calculation and prediction of AD neuropathology in the test data

With the selected variables from Stage 2, PS are calculated in the test dataset.

Three different PS were calculated (after equation (1)): Single-omics PS (PS_DNAm_; PS_Metabolome_) that only contain information from one omics layer (either DNAm or metabolomics), multi-omics PS that contain both the DNAm and metabolomics data (PS_DNAm+Metabolome_), and joint PS models that contain the individual single-omics PS (PS_DNAm_; PS_Metabolome_) in the same prediction model with and without an interaction term between the single-omics PS (PS_DNAm_ * PS_Metabolome_). In our analysis, the outcome is ordinal, and all three models are ordinal logistic regression models [51, 52] with the following equations:

(2) $$\text{Single-omics}\;\text{PS}:\;\text{Neurop.}\;\text{outcome}\sim PS_{\text{DNAm}}\left(\text{or}\;\right.\left.PS_{\text{Metabolome}}\right)+\text{Covariates},$$

(3) $$\text{Multi-omics}\;\text{PS}:\;\text{Neurop.}\;\text{outcome}\sim PS_{\text{DNAm}+\text{Metabolome}}+\text{Covariates},$$

(4) $$\text{Joint}\;\text{PS}\;\text{model}:\;\text{Neurop}.\text{outcome}\sim PS_{\text{DNAm}}+PS_{\text{Metabolome}}\left(+PS_{\text{DNAm}}*PS_{\text{Metabolome}}\right)+\text{Covariates}.$$

#### Stage 4 - Validation of PS models in the test data

The performance of the single-, multi-omics and joint PS models was evaluated in the test data based on a partial McFadden’s R^2^, also known as the partial pseudo-R^2^, which is a measure of goodness of fit for logistic regression models, including ordinal logistic regression (can be found in the Supplementary Materials). Calculating partial R^2^ allowed us to demonstrate the prediction R^2^ for the PS, independent of the influence of the other covariates (sex, race, educational attainment, age at death, ADI, PMI). We further evaluated whether the PS was significantly associated with the neuropathology outcomes in the independent test data using a likelihood ratio test (p-value<0.05) in the ordinal logistic regression models from Step 3.

#### Stage 5 - Pathway analysis of selected metabolites and/or CpG sites

The final step involves a pathway analysis of the identified omics variables (CpG site and/or metabolomics features). Pathway analysis maps selected CpG sites and/or metabolomics features to known biological pathways, elucidating the biological mechanisms potentially underlying observed associations with neuropathology. Only the best performing PS were used for the pathway analyses. We included the features/CpG sites that were selected in at least one, two, or three of the 10 iterations in the pathway enrichment analysis.

For the DNAm PS, we conducted gene set enrichment analyses using the R package missMethyl [25, 53–55] and the KEGG database [56–58]. For the metabolomics PS, datasets for positive and negative ion modes were merged with experimental results to match mass-to-charge ratios (m/z) and retention times. Next, we used the R package metapone [59] to identify pathways from the KEGG database associated with the detected metabolites by leveraging adduct information and permutation-based statistical thresholds.

Shared pathways between both omics layers were identified and their respective p-values were combined using the Fisher method for p-value combination [60, 61]. To visually compare the pathways across the two omics layers, −log10-transformed p-values from the DNA methylation analysis were plotted against those from the metabolomics analysis, and point color indicated the combined pathway significance.

##### Gene–metabolite network analysis

To explore potential relationships between molecular layers, a gene–metabolite interaction network was constructed and visualized using Cytoscape (version 3.10.1). Significant genes and metabolites identified from the statistical analyses were integrated into a bipartite network based on their pairwise associations. The resulting network was then examined to identify clusters of interconnected genes and metabolites, highlighting potential functional relationships and key molecular hubs within the multi-omics dataset.

#### Sensitivity Analyses

While the main analyses were conducted for the largest sample possible (N=154 for PS_DNAm_, N=141 for PS_Metabolome_, N=138 for the multi-omics PS and the joint PS model), we conducted a sensitivity analysis, in which we restricted the PS_DNAm_ and PS_Metabolome_ to the donors with data on DNAm and metabolomics (N=138), to validate that differences between the different PS models are not due to differences in sample size.

#### Comparison of PS results to Multi-Omics Factor Analysis (MOFA)

To compare the results from our PS analysis to one of the most established multi-omics approaches, we conducted Multi-Omics Factor Analysis (MOFA), an unsupervised dimensionality reduction approach to identify latent factors explaining variability across multiple high-dimensional omics datasets simultaneously [62] Such latent factors enable detecting common as well as unique data type–specific patterns and revealing coordinated biological signals across molecular layers. MOFA enables interpretation of complex multi-omics relationships by projecting the original data into a reduced latent space and emphasizes important drivers of molecular variation. The approach has been tailored to conducting more integrative analyses of heterogeneous multi-omics data [62].

## Results

### Study population

After excluding 4 brain donors with missing covariate information, a total of 157 samples were included in the current analysis (Table 1). 154 of them had DNAm data available, 141 of them had metabolomics data available and 138 of them had both DNAm and metabolomics data available.

The mean age at death was 76.4 years (standard deviation [SD]: 10.0). Most participants were White (89.2%) and 10.8% self-identified as Black or African American. The study population consisted of 54.8% males and 45.2% females. The study population was predominantly well-educated with 49.7% holding a college degree, and 28.0% a graduate degree. The mean ADI score was 36.1, with a standard deviation of 24.0, indicating a wide range in socioeconomic deprivation among the participants. The prevalence of at least one APOE ε4 allele (56.1%) was much higher than that in the general population in the United States, which is estimated to be around 20–30% [63]. Most donors (59.2%) had high levels of AD neuropathologic changes (ABC score of “high”), 47.1% of donors were classified as Braak Stage 6 and 70.1% of donors had frequent neuritic plaques on the CERAD score, indicating a high prevalence of AD neuropathology in this study population.

### Single-omics PS

**Figure 2.**
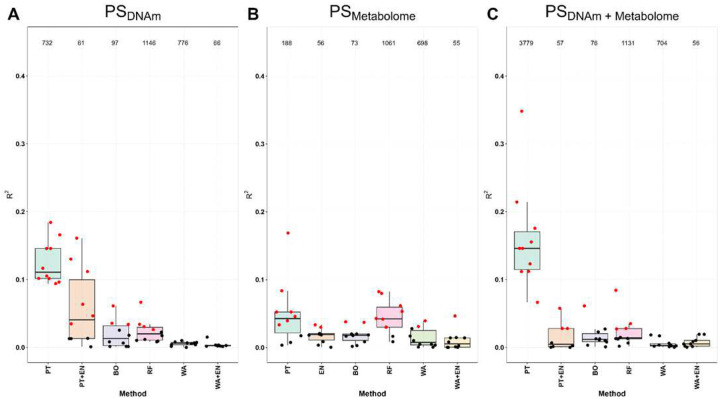
Overview of the results of PS calculation using the various methods and their accuracy of fitting from the individual and combined data sets (McFadden *R*2 and p-value) on ABC score outcome. All three sub-graphs are structured in the same way and therefore the explanation can be made on one graph and apply to all three graphs: A) DNAm data set (single-omics PS), B) metabolome data set (single-omics PS) and C) combination of both individual data sets (multi-omics PS). The following 6 methods are shown on the x-axis: PT: Pruning & Thresholding, EN: Elastic Net or EN+PT, BO: Boosting, RF: Random Forest, WA: Windows Approach and WA+EN (due to the high dimension, EN can only be applied to the smaller data set and then PT is used before for dimension reduction). Partial McFadden R2 is shown on the y-axis, so that for each method a boxplot is shown for the 10 iterations, with the individual results shown as black (not significant) and red (significant) points. At the top are the recorded number of features or CpG-sites included in the PS (mean value across the 10 iterations).

First, we calculated single-omics PS (equation (1)) based on DNAm and metabolomics data (Figure 1). PS_DNAm_ and PS_Metabolome_ were calculated for each of the three neuropathological outcomes (ABC score, Braak stage and CERAD score) using six different approaches (PT: Pruning & Thresholding, EN: Elastic Net, BO: Boosting, RF: Random Forest, WA: Windows Approach). The results for the ABC score are shown in Figure 2 and the results for the other two outcomes are included in the appendix (Figures S1 – S4). The results from all three outcomes were similar.

For the PS_DNAm_ (Figure 2A), PT reaches the highest median R^2^ (0.11) over all other methods and found a significant association between the DNAm PS and the ABC score in all 10 iterations. Compared to the PT approach, the RF approach performed worse in terms of both the median R^2^ value of 0.02 and the number of significant associations (4 out of 10). Other noteworthy approaches for the DNAm PS are PT+EN and BO. The median R^2^ was similar to the RF approach (PT+EN: 0.05; BO: 0.02) and PT+EN identified a significant association between DNAm PS and ABC score in 6 out of 10 iterations and the BO approach in 3 out of 10 iterations. WA and WA+EN resulted in a smaller median R^2^ and no significant associations with the ABC score.

For the PS_Metabolome_ (Figure 2B), the RF approach performed best, leading to a median R^2^ value of 0.04 across the 10 iterations, identifying a significant association between the metabolomics PS and the ABC score in 8 out of 10 iterations. While the PT approach detected a significant association in 7 out of 10 iterations, its median R^2^ value was similar to the RF approach (R^2^=0.04). The other approaches resulted in fewer significant associations and lower R^2^ values.

The results were similar in a sensitivity analysis, in which we restricted the PS_DNAm_ and PS_Metabolome_ to the donors with data on DNAm and metabolomics (N=138; Supplementary Figure S6).

### Multi-omics PS

When combining the DNAm and metabolomics data in one dataset to derive a multi-omics PS (PS_DNAm+Metabolome_; equation (3)), the PT approach (R^2^=0.15, 10 out of 10 significant associations) performed best, followed by the RF approach (R^2^=0.01, 2 out of 10 significant associations) (Figure 2C). The R^2^ values for the PT approach were greater than in the single-omics PS (Figure 2A & 2B).

### Joint PS models

**Figure 3.**
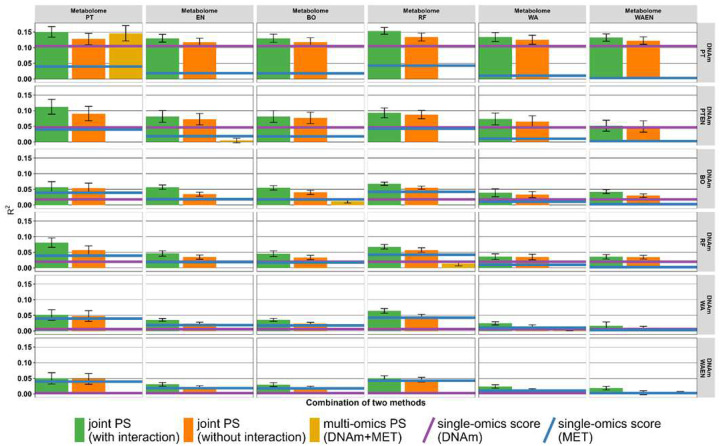
Results from single- and multi-omics PS each individual graph shows the R2 for the PS of the individual and combined datasets, with the method from the DNAm dataset shown on the x-axis and the metabolome dataset on the y-axis (ABC score). The methods are PT: Pruning & Thresholding, EN: Elastic Net, BO: Boosting, RF: Random Forest, WA: Windows Approach and WA+EN. Partial McFadden R2 is shown on the y-axis, so that for each method the median with standard error (SE) is drawn as bar plot with SE bars. The values in a subgraph are from the following data sets or combinations: Joint PS models with interaction term (green), Joint PS models without interaction term (orange) and multi-omics PS from both combined data sets (golden). Since the single PS are at most as good (median) as the joint PS, we have omitted these for better consideration and drawn only the PS with the highest value as a line in the subgraph. If the single PS from DNAm data set is higher than the PS from the metabolome data set, we took the value of the DNAm PS and drew a blue line (vice versa for the single PS from metabolome data set purple). Due to the calculation, the golden boxplots are only present in the subgraphs that use the same methods in both data sets.

Next, we included the individual DNAm PS and metabolomics PS in a joint PS model (with and without an interaction term between the two PS; equation (4)) to evaluate whether this results in a higher model prediction performance (Figure 3).

For the ABC score, the best model R^2^ (0.15) was reached when combining the DNAm PS based on the PT approach with the metabolomics PS based on the RF approach, which were also identified as the best PS in the individual omics data (Figure 3). R^2^ for the model with the interaction term (R^2^=0.15) was slightly higher than R^2^ for the model without the interaction term between the two PS (R^2^=0.13). Across methods, joint PS models with interaction generally yielded higher R^2^ values than models without interaction, while multi-omics PS showed heterogeneous performance and exceeded the joint models only for the PT method.

Detailed results for the ABC score, Braak stage, and CERAD score are provided in the Supplementary Material (Tables ST1–ST3). The results from all three outcomes were similar.

### Sensitivity analyses

When restricting the sample size to the samples with DNAm and metabolomics data (N = 138), the previously identified best-performing approaches (PT for DNAm and RF for metabolome) demonstrated similar R^2^ values to the main single-omics analyses, with medians closely aligning to those obtained from the full datasets (see Supplement Figure S5).

When conducting 100 instead of 10 iterations, the joint PS models—with and without interaction terms—exhibited similar performance in terms of both the number of captured features/CpG sites and R^2^, with results largely consistent with the mean estimates from the main analyses (Supplement Figure S6).

### Secondary analyses of the best-performing PS

The two best-performing single-omics PS (PT for DNAm and RF for metabolomics) showed a Pearson correlation of = 0.25) (see Supplement Figure S7). Correlations between the other single-omics PS were lower ranging from 0.00 to 0.19.

#### Pathway enrichment analysis

To determine the biological significance of the features included in our PS, we carried out pathway enrichment analyses for the features included in the DNAm and metabolomics PS separately (see Supplement Figure S8, S9). KEGG pathway enrichment examination revealed several pathways that have been linked to lipid metabolism and signal transduction in both the omics layers. For the DNAm-based profile scores, enriched pathways included lipid metabolism pathways and several signaling pathways previously linked to Alzheimer’s disease. Metabolomics enrichment analysis also underscored perturbations in lipid-related metabolic pathways and signal transduction mechanisms associated with AD related neuropathology.

#### Cross-omics pathway integration

**Figure 4.**
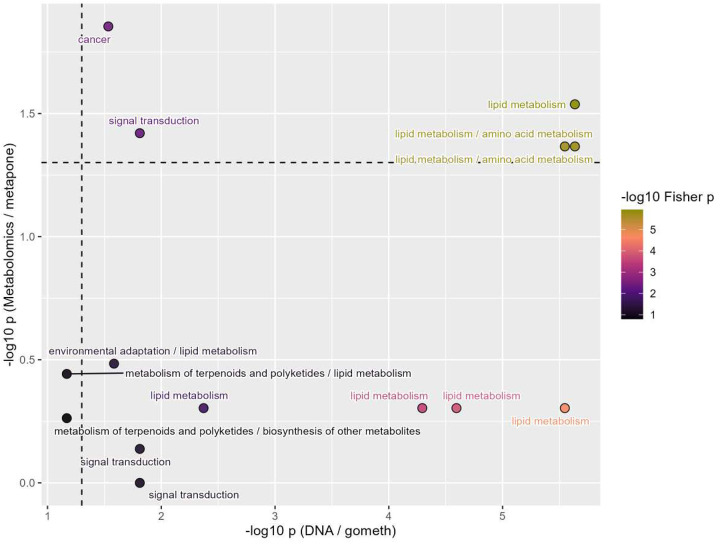
Scatter plot showing pathway enrichment results derived from DNA methylation and metabolomics datasets. The x-axis represents −log10 transformed p-values from the methylation-based pathway enrichment, whereas the y-axis shows −log10 transformed p-values from metabolomics enrichment analysis. Each point corresponds to a biological pathway, labeled by its functional category. The color scale indicates the significance level based on Fisher’s test (−log10 p-value) for combing p-values. Dashed lines represent the significance threshold (p = 0.05) for both analyses. Pathways located in the upper-right quadrant indicate concordant enrichment in both omics layers, highlighting lipid metabolism–related pathways as jointly affected.

To assess biological correlation between the two single-omics PS, pathway enrichment results from DNAm (gometh) and metabolomics (metapone) analyses were integrated. Shared pathways were matched and p-values were pooled according to Fisher’s method (Figure 4). Most pathways were only identified in a single omics layer, suggesting that DNA methylation and metabolomics capture partially distinct aspects of Alzheimer’s disease biology. Shared pathways across both omics layers included lipid metabolism like alpha-linolenic acid metabolism, linoleic acid metabolism, and arachidonic acid metabolism.

#### Gene–metabolite network analysis

**Figure 5.**
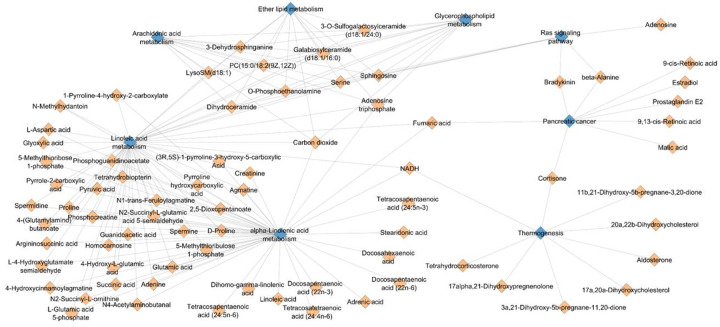
Network visualization illustrates the relationships between significantly associated metabolites and metabolic pathways. Diamond-shaped nodes represent metabolites, while blue nodes denote metabolic pathways. Edges indicate biochemical or pathway-based associations between metabolites and pathways derived from enrichment analysis. Highly connected hubs correspond to lipid-related pathways such as either lipid metabolism, glycerophospholipid metabolism, and arachidonic acid metabolism, suggesting a central role of lipid metabolism in the observed metabolic alterations.

We then constructed a gene–metabolite interaction network using HMDB metabolite annotations and KEGG pathway mappings to investigate further functional relationships between epigenetic and metabolic signals. The network established (Figure 5) demonstrates multiple biologically consistent clusters that connect metabolites to the enriched pathways identified in the DNA methylation analysis. In particular, several metabolites converge on lipid-related pathways such as alpha-linolenic acid metabolism, arachidonic acid metabolism, and glycerophospholipid metabolism. These pathways act as central hubs within the network and connect multiple metabolites simultaneously, indicating that lipid metabolism may epitomize an important interface between epigenetic regulation and metabolic changes in the context of Alzheimer’s disease. Furthermore, some metabolites are found to link to multiple pathways, suggesting potential metabolomic cross-talk between different biological processes. This network pattern demonstrates potential regulatory relationships between epigenetic signals and the subsequent metabolic pathways and offers a systems-level representation of molecular interactions associated with Alzheimer’s disease neuropathology. These network findings reinforce our cross-omics pathway integration results, which also revealed lipid metabolism as a pathway consistently supported by both omics layers. Collectively, the connectivity pattern emphasizes lipid metabolism as a central molecular hub that connects several metabolites to pathways enriched in epigenetics. This observation is consistent with the premise that lipid-related metabolic processes are an important crossroads of epigenetic regulation and downstream metabolic changes in Alzheimer’s disease.

### Comparison of PS results to Multi-Omics Factor Analysis (MOFA)

We conducted MOFA to compare the results from our PS analysis to one of the most established multi-omics approaches (see Supplement Figure S10). The analysis showed that most latent factors were predominantly explained by variation in a single omics layer. No significant latent factor was identified explaining a large portion of variance across both omics layers simultaneously. These results indicate that DNA methylation and metabolomics provide largely distinct biological signals in these brain tissue samples. This observation is in line with the comparably small predictive gains when two omics layers are employed together in the multi-omics profile score models. Interestingly, pathway-based analysis identified several overlapping biological pathways across data modalities that were not captured by MOFA. This suggests that shared biological signals may exist but are not represented as dominant sources of variance in the latent factor model.

Taken together, these findings indicate that DNAm is the primary driver of predictive performance, while metabolomics provides complementary but modest incremental value in this modeling framework.

## Discussion

We performed a comprehensive single- and multi-omics analysis integrating genome-wide DNAm and high-resolution metabolomics data derived from 157 frontal cortex samples, aiming to gain a better understanding of AD neuropathology. We developed single- and multi-omics PS based on DNAm and metabolomics data to predict the neuropathological features of AD independent of age, sex, race and socioeconomic factors, using various machine learning and regression-based approaches. The best-performing PS_DNAm_, which was calculated using the PT approach, predicted AD neuropathology levels with a median partial R^2^ of 0.11 and the best-performing PS_Metabolome_, which was calculated using RF, reached a median R^2^ of 0.04. The best joint PS model which combined the DNAm and metabolomics data led to an increase in predictive accuracy (R^2^ = 0.15). The moderate correlation between PS_DNAm_ and PS_Metabolome_ (r=0.25) suggests that the two omics layers capture largely complementary biological signals rather than redundant information. This observation is consistent with the MOFA results, which indicated that most latent factors were omics layer-specific. While MOFA did not identify joint biological pathways, cross-omics pathways integration and gene-metabolite network analysis of the features identified by PS_DNAm_ and PS_Metabolome_ revealed lipid metabolism and signal transduction as joint biological pathways across both omics layers.

Our analysis showed that DNAm-based PS had a better predictive performance for AD neuropathology than metabolomics-based PS. The DNAm PS achieved a median R^2^ value of 0.11, while the best metabolomics PS only achieved a median R^2^ value of 0.04. In all ten iterations, the DNAm PS showed significant associations with the ABC score, while the metabolomics PS produced significant results in fewer iterations. These results suggest that DNAm may be a stronger indicator of neuropathologic changes in AD than metabolomics. Current evidence highlights the distinct yet complementary roles of brain DNAm and metabolomics in understanding neuropathology markers associated with AD. Studies have shown that alterations in DNAm patterns are closely linked to neuroinflammation, oxidative stress, and other pathological processes in AD. For instance, global hypomethylation and gene-specific methylation changes have been identified as significant contributors to disease progression, impacting genes involved in synaptic function and neurodegeneration [8]. On the other hand, brain metabolomics has recently emerged as a powerful tool for identifying metabolic dysfunctions associated with AD. Research indicates that specific metabolic pathways—such as those involving lipid metabolism, energy production, and amino acid metabolism—are significantly altered in the brains of individuals with AD. For example, dysregulation of metabolites related to energy metabolism and lipid profiles has been correlated with cognitive decline and neuropathological markers like amyloid plaques and neurofibrillary tangles [10, 11].

A small proportion of biological pathways were consistently identified across both omics layers. Many lipid metabolism pathways, in particular alpha-linolenic acid metabolism, linoleic acid metabolism, and arachidonic acid metabolism, were enriched across both omics layers. Many of the overlapping pathways play significant roles in AD pathology. For instance, the alpha linolenic acid and metabolism and linoleic acid metabolism have been previously linked with AD [64–66]. Alpha-linolenic acid is an essential omega-3 fatty acid known for its anti-inflammatory properties and its role in maintaining neuronal health. Studies have shown that an imbalance in fatty acid metabolism, including alpha-linolenic acid, is linked to neurodegenerative diseases such as AD [64, 65]. Linoleic acid, on the other hand, is an essential omega-6 fatty acid whose dysregulation has also been associated with AD. It influences inflammatory processes and can lead to the formation of bioactive lipids that are important for neuronal health [66]. These results highlight that an integrated analysis of both omics data can contribute to improving the understanding of disease mechanisms.

In addition to the two shared pathways, CpG sites selected by the PS_DNAm_ were mapped to eight additional AD-related pathways that were unique for the DNAm data. The pentose phosphate pathway is crucial for maintaining redox balance in the brain through nicotinamide adenine dinucleotide phosphate production, which reduces oxidative stress—a key factor in AD [67] .Dysregulated glycerophospholipid metabolism contributes to membrane dysfunction, which is linked to amyloid-beta and tau pathology in AD [68]. Ether lipids are essential for neuronal function, and their dysregulation has been observed in neurodegenerative diseases, including AD [69]. Arachidonic acid and its metabolites regulate inflammation and oxidative stress, both of which are implicated in AD [70]. Linoleic acid is a precursor to bioactive lipids that influence inflammation and neuronal health. Its dysregulation has been linked to AD [71]. Dysregulation of Ras signaling affects synaptic plasticity and memory, both of which are impaired in AD [72]. The complement system is involved in neuroinflammation and amyloid clearance in AD. Dysregulation contributes to neurodegeneration [73]. Impaired thermogenesis may contribute to metabolic dysfunction observed in AD [74].

For the metabolomics data, ten unique pathways were identified with a known link to AD. Arginine metabolism is closely involved in systemic inflammation and can influence neuroinflammation, which plays a crucial role in AD. Proline is associated with oxidative stress, another key factor in AD pathology [75]. Dysregulation of sphingolipids has been linked to amyloid-beta aggregation and tau pathology, both hallmarks of AD [66]. Steroid hormones, such as estrogens and androgens, have neuroprotective properties. Their decline in aging is associated with AD progression [76]. Cholesterol levels affect the production and aggregation of amyloid-beta peptides, contributing to AD pathology [77]. Neurosteroids derived from C21 steroids are known to modulate inflammation and neuronal health in AD [78]. Erythropoietin (EPO) has been shown to exhibit neuroprotective effects in preclinical AD models [79]. Insulin resistance in the brain is a hallmark of AD and is sometimes referred to as “Type 3 diabetes” [80]. Adenosine A2A receptors modulate neuroinflammation and synaptic plasticity, both implicated in AD progression [81]. Drug metabolism impacts the effectiveness of AD therapies, which can influence disease progression [82]. Cytochrome P450 enzymes are involved in the metabolism of amyloid-beta and oxidative stress pathways in AD [83].

Among the statistical methods used, there were clear differences in the performance for different omics data. For the DNAm data, PT was the best-performing method based on the R^2^. For the metabolomics data, RF produced the highest R^2^. While extensive simulation studies are needed to explain the observed differences in performance for different omics data, it could be due to the different number of variables in DNAm (789,286 CpG sites) versus metabolomics (35,348 variables) data or differences in the distribution, correlation structures, or underlying interactions between variables. In comparison, EN and WA led to a substantially lower R^2^ for both omics layers and EN also clearly underperformed in terms of computational time. WA was by far the fastest method. BO showed a similar performance as EN, but future studies need to determine whether an improved hyperparameter optimization could increase the performance of this method. Of note, we mainly focused on the predictive performance for the model comparison. If interested in association testing, researchers should focus on the joint PS model without an interaction term to receive valid effect estimates for each single-omics PS.

In our study, we developed novel brain tissue–based multi-omics profile scores for AD neuropathology that integrate genome-wide DNAm and high-resolution metabolomics data, revealing that combining these omics layers modestly improves predictive accuracy compared to using either layer alone. This finding aligns with a growing body of literature exploring individual omics associations with AD-related outcomes. Polygenic risk scores, which are based on genotype data, have been developed and tested for regional brain volume differences [84] neurocognitive test scores [85], and dementia-related blood biomarker levels [86]. Leonenko et al. [86] refined risk prediction by showing that modeling APOE separately from the polygenic risk score enhances its predictive power, and Wang et al. [87] used unsupervised machine learning to develop an AD risk score that integrates cerebrospinal fluid, MRI, and cognitive data, highlighting the utility of multi-modal approaches. Moreover, Koetsier et al. [88]developed a blood-based DNAm risk score to predict cognitive impairment and dementia, directly supporting the role of epigenetic alterations in AD pathology, while Cary et al. [89] further advanced the field by integrating genetic, transcriptomic, and proteomic data to map AD risk onto core biological domains such as synapse function, immune response, and lipid metabolism. In a study by Liu et al. [90], comprehensive genetic prediction models were developed to compute risk scores for blood metabolites, which were subsequently employed in association analyses with Alzheimer’s disease risk. Together, these studies emphasize that several omics layers are individually associated with AD-related outcomes, and they provide a valuable context for our results which show that the integration of these omics layers can offer deeper insights into the molecular underpinnings of AD.

There are several strengths of our study to be noted. The unique dataset includes both well-characterized DNAm and metabolomics data from 138 brain donors, allowing a detailed examination of brain-based multi-omics profiles related to AD neuropathology. This combination provides the opportunity to identify potential biomolecular markers that may be crucial for understanding disease mechanisms. Our study is characterized by methodological advances that enable a comprehensive analysis of DNAm and metabolomics. This paper presents a comprehensive multi-omics analysis that integrates both DNA methylation data and high-resolution metabolomics data using profile scores. This innovative approach allows for a better understanding of biological mechanisms associated with AD. While previous studies often analyzed isolated omics data independently (e.g., only DNA methylation or only metabolomics), this analysis combines both types of data to obtain a holistic picture of biological processes. Pathway enrichment analysis was used to identify common biological pathways that may not have been previously recognized and that could not be detected using other multi-omics approaches (e.g., MOFA). Overall, these methodological improvements facilitate a deeper understanding of the intricate relationships between epigenetic modifications and metabolic changes in Alzheimer’s disease pathology.

In addition to its strengths, our study has some limitations that should be considered. One notable aspect is that the ADRC cohort is enriched with cases AD and other dementias, which makes the brain bank a convenience sample rather than a population-based one. This concentration of AD cases may reduce variability in neuropathology markers within the sample. Another consideration is the relatively small sample size when splitting our data into training and test sets to prevent overfitting. However, it is important to highlight that few studies have access to such a large autopsy sample, which is crucial for accurately measuring neuropathology markers as well as brain tissue-based DNAm and metabolomics data—this represents a significant strength of our work. While integrating DNAm and metabolomics has improved predictive power, further exploration is needed to understand how these epigenetic and metabolic changes interact and jointly influence AD pathology. The use of postmortem tissue samples also introduces some limitations; they may not fully capture dynamic metabolic and epigenetic changes throughout disease progression, potentially overlooking important temporal variations in biomarkers. Additionally, applying machine learning methods such as Pruning & Thresholding, Random Forests, Elastic Net, Boosting, and Sliding Windows can present challenges related to hyperparameter selection and optimization. While a general optimization of the hyperparameter was performed and used in each model, future work should evaluate whether these factors can be further optimized to improve the model performance and the robustness of analyses.

Future research directions should focus on expanding the sample size and diversity 3to validate the results and ensure their generalizability. Integrating additional levels of omics such as genomics, proteomics, transcriptomics, lipidomics, and microbiome could provide a more comprehensive view of the pathophysiology of AD. Exploring interactions between these different layers could help to identify novel biomarkers and signaling pathways.

## Conclusion

In conclusion, our findings show that the integration of DNAm and metabolomics data in a multi-omics profile score framework is feasible for investigating the neuropathology of Alzheimer’s disease. Although the improvement in predictive performance was modest, the integrative analyses consistently highlighted biologically relevant pathways, particularly lipid metabolism and signal transduction, across epigenetic, metabolic, and network levels. Future studies incorporating larger and more diverse cohorts and additional omics layers will be important to further validate these findings and to advance the translation of multi-omics insights into a deeper understanding of the pathophysiology of Alzheimer’s disease. ***

## Supplementary Material

Supplementary Files

This is a list of supplementary files associated with this preprint. Click to download.

- Supplementfiles.docx

## Figures and Tables

**Table 1.** Characteristics of the study population.

	Total (N=157)	DNAm (N=154)	Metabolomics (N=141)	Multi-omics analyses (N=138)
**Age at Death**				
Mean (SD)	76.4 (10.0)	76.4 (10.0)	76.7 (10.2)	76.7 (10.2)
Range	57.0 – 105.0	57.0 – 105.0	57.0 – 105.0	57.0 – 105.0
**Race**				
White	140 (89.2%)	137 (89.0%)	124 (87.9%)	121 (87.7%)
Black	17 (10.8%)	17 (11.0%)	17 (12.1%)	17 (12.3%)
**Sex**				
Female	71 (45.2%)	70 (45.5%)	61 (43.3%)	60 (43.5%)
Male	86 (54.8%)	84 (54.5%)	80 (56.7%)	78 (56.5%)
**Post Mortal Index (PMI) (in hours)**				
Mean (SD)	11.6 (9.6)	11.6 (9.6)	11.5 (9.7)	11.5 (9.7)
Range	1.5 – 64.0	1.5 – 64.0	1.5 – 64.0	1.5 – 64.0
**Education (cat.)**				
High school or less	35 (22.3%)	35 (22.7%)	29 (20.6%)	29 (21.0%)
College degree	78 (49.7%)	76 (49.4%)	70 (49.6%)	68 (49.3%)
Graduate degree	44 (28.0%)	43 (27.9%)	42 (29.8%)	41 (29.7%)
**Area Deprivation Index (ADI)**				
Mean (SD)	36.1 (24.0)	36.3 (24.1)	34.4 (23.7)	34.6 (23.9)
Range	1.0 – 94.0	1.0 – 94.0	1.0 – 94.0	1.0 – 94.0
**APOE (yes/no)**				
E4 absent	69 (43.9%)	69 (44.8%)	60 (42.6%)	60 (43.5%)
E4 present	88 (56.1%)	85 (55.2%)	81 (57.4%)	78 (56.5%)
**ABC score**				
Not	15 (9.6%)	15 (9.7%)	13 (9.2%)	13 (9.4%)
Low	28 (17.8%)	28 (18.2%)	24 (17.0%)	24 (17.4%)
Intermediate	21 (13.4%)	20 (13.0%)	20 (14.2%)	19 (13.8%)
High	93 (59.2%)	91 (59.1%)	84 (59.6%)	82 (59.4%)
**Braak Stage**				
Stage 0	0 (0.0%)	0 (0.0%)	0 (0.0%)	0 (0.0%)
Stage 1	16 (10.2%)	16 (10.4%)	15 (10.6%)	15 (10.9%)
Stage 2	11 (7.0%)	11 (7.1%)	10 (7.1%)	10 (7.2%)
Stage 3	17 (10.8%)	17 (11.0%)	13 (9.2%)	13 (9.4%)
Stage 4	18 (11.5%)	17 (11.0%)	17 (12.1%)	16 (11.6%)
Stage 5	21 (13.4%)	21 (13.6%)	19 (13.5%)	19 (13.8%)
Stage 6	74 (47.1%)	72 (46.8%)	67 (47.5%)	65 (47.1%)
**CERAD score**				
No	34 (21.7%)	34 (22.1%)	30 (21.3%)	30 (21.7%)
Sparse	3 (1.9%)	3 (1.9%)	3 (2.1%)	3 (2.2%)
Moderate	10 (6.4%)	10 (6.5%)	9 (6.4%)	9 (6.5%)
Frequent	110 (70.1%)	107 (69.5%)	99 (70.2%)	96 (69.6%)

Donors included in the column “total” had information on all covariates, neuropathology outcomes and either DNAm or metabolomics data available. Of these, 154 donors (listed in the column “DNAm” had DNAm available and 141 donors (listed in the column “metabolomics”) had metabolomics data available. Donors included in the column “multi-omics analyses” had data on DNAm and metabolomics and were included in the multi-omics analyses.

## Data Availability

Requests for data access should be submitted through the Alzheimer’s Disease Research Center at Emory University via the following link: https://alzheimers.emory.edu/research/for-researchers/data-request-form.html.

## List of abbreviations

ABC: Amyloid, Braak, CERAD
AD: Alzheimer’s disease
ADI: Area Deprivation Index
ADRC: Alzheimer’s Disease Research Center
BO: Boosting
CERAD: Consortium to Establish a Registry for Alzheimer’s Disease
CpG: cytosine-phosphate-guanine
DNAm: DNA methylation
EN: Elastic Net
HILIC: hydrophilic interaction liquid chromatography
LC-HRMS: liquid chromatography coupled with high-resolution mass spectrometry
MOFA: Multi-Omics Factor Analysis
m/z: mass-to-charge ratio
NFTs: neurofibrillary tangles
PMI: postmortem interval
PS: Profile score(s)
PT: Pruning & Thresholding
RF: Random Forest
RT: retention time
SD: standard deviation
SNP: single nucleotide polymorphism
VIMs: variable importance measures
WA: Windows Approach
